# Impact of physical activity on the incidence of psychiatric conditions during childhood: a longitudinal Swedish birth cohort study

**DOI:** 10.1136/bjsports-2024-108148

**Published:** 2025-05-13

**Authors:** Oskar Lundgren, Hanna Tigerstrand, Andrea Lebena, Marie Löf, Johnny Ludvigsson

**Affiliations:** 1 Division of Pediatrics, Department of Biomedical and Clinical Sciences, Linköping University, Linköping, Sweden; 2 Crown Princess Victoria Children’s Hospital, Linköping University Hospital, Linköping, Sweden; 3 Division of Physiotherapy, Department of Health, Medicine and Caring Sciences, Linköping University, Linköping, Sweden; 4 Department of Medicine Huddinge, Karolinska Institute, Stockholm, Sweden

**Keywords:** Physical activity, Sports, Child Health, Psychiatry

## Abstract

**Background:**

Emerging evidence supports the importance of physical activity (PA) and behaviours that build resilience to prevent childhood psychiatric disorders.

**Objective:**

To investigate associations between parent-reported PA, time spent outdoors and participation in organised sports and later incidence of psychiatric conditions in children.

**Methods:**

A birth cohort of 17 055 Swedish children was followed up until age 18 years. Data on parent-reported PA, time outdoors and participation in organised sports were collected for children at ages 5, 8 and 11. Diagnoses of psychiatric conditions were obtained from a national registry. Longitudinal interactions were analysed with two-way analysis of variance, and hazard ratios for incidence until 18 years were calculated with Cox proportional hazard models, adjusting for mothers’ education and use of psychotropics, children’s adverse life events and sex.

**Results:**

PA declined from 4.2 to 2.5 hours per day between 5 and 11 years of age. PA at 11 years was negatively associated with the incidence of any psychiatric conditions among all participants (HR=0.88, 95% CI 0.79 to 0.98) until 18 years. PA at 11 years showed a trend for reducing depression among girls (HR=0.82, 95% CI 0.67 to 1.00) and boys (HR=0.71, 95% CI 0.47 to 1.06) and protected against anxiety (HR=0.61, 95% CI 0.42 to 0.90) and addiction (HR=0.65, 95% CI 0.45 to 0.95) for boys, but not for girls (anxiety: HR=0.96, 95% CI 0.81 to 1.13, addiction: HR=1.04, 95% CI 0.68 to 1.58). Time outdoors showed no protective associations, while participation in organised sports showed significant protective effects on anxiety and addiction for both boys and girls, and on depression for boys.

**Conclusions:**

This study provides evidence that PA and participation in organised sports may have sex-specific protective effects against several childhood psychiatric conditions.

**WHAT IS ALREADY KNOWN ON THIS TOPIC**

- Previous studies have explored the longitudinal association between physical activity (PA) and psychiatric disease in childhood, with most reporting a negative association between PA and the incidence of depression and anxiety disorders.
- Furthermore, existing meta-analytic data have supported the beneficial effect of PA interventions on psychosocial outcomes across different age groups.
- However, many studies had short follow-up times, focused on a limited part of childhood, and the strength of associations has varied, depending on the target population and psychosocial condition studied.
- The great diversity in methods for the assessment of psychiatric symptoms, and variability in the assessment of PA, has made comparisons difficult and lowered the quality of evidence.

**WHAT THIS STUDY ADDS**

- This large, longitudinal birth cohort study, representative of the Swedish general population, with 18 years of follow-up, showed that PA declines from 5 to 11 years of age and provides sex-specific protective effects against the incidence of several psychiatric diseases up to the age of 18.
- PA at 11 years of age had a protective effect on the incidence of any psychiatric disease for boys, and on depression among girls.
- Participation in organised sports showed strong protective effects for both girls and boys on multiple outcomes, while time outdoors showed no associations.
- Our study suggests that pre-adolescence, in addition to being a critical period for the emergence of psychiatric disorders, may also be a sensitive period during which PA is important to prevent the development of psychiatric disorders in children and adolescents.

**HOW THIS STUDY MIGHT AFFECT RESEARCH, PRACTICE OR POLICY**

- Given the dramatic increase in the global prevalence of psychiatric disorders among children and adolescents, this study highlights the importance of promoting PA, particularly through organised sports.
- PA could be used as a strategy for the prevention of mental health problems, especially before, and during, adolescence, and should spark increased interest among policymakers and motivate healthcare professionals to incorporate PA in treatment programmes.
- Further identifying the mechanisms involved, optimal timing of interventions, and the role of comorbidities and social factors, should be research priorities.

## Introduction

 With a high global prevalence of mental disorders among children and adolescents and a dramatic increase during the last decades, the need for prevention strategies is obvious.^1^ The rise in mental disorders is associated with both increased costs,^2^ and individual impact, including an increased risk for suicide.^3^ Our knowledge of why some individuals develop mental disorders is far from complete. Several theories have been proposed, and one of the most common is the stress-vulnerability model.^4^ The model suggests that a genetic predisposition to stress interacts with other factors in the development of psychiatric disease. Although the model has been criticised for being overly deterministic,^5^ it highlights the interplay between the individual and the environment, and investigations have revealed important pathways for the way in which stress translates into dysfunction, including in the endocrine system^6^ and the immune system.^7^ However, a focus on vulnerability comes with the risk of neglecting the various ways in which humans cope with difficulties. Some researchers have instead proposed a resilience framework to invigorate the development of new ways to prevent and treat mental disorders.^8^

Physical activity (PA) has been suggested to be a key resilience factor for lowering the risk of the development of mental disorders among children and adolescents. PA reduces inflammation, increases resilience to stress and might also have a positive effect on self-esteem, which in turn can influence symptoms of mental illness.^9^ Meta-analytic evidence has supported the suggestion that PA can improve mental health in both children and adults.^10^ Schuch and Vancampfort recently declared that the evidence is sufficiently substantial for PA to be used as both a treatment and for prevention against psychiatric disease.^11^ However, other authors have argued that the evidence is still unconvincing.^12^

An important question is whether there are time points during childhood when PA plays a more central role in the development of mental disorders.^10^ There are few studies with more than a few years of follow-up.^13^ A notable exception is the Raine birth cohort of 1628 Australian children, whose trajectories of PA and self-reported mental well-being were assessed repeatedly until early adulthood, and children with high PA reported a favourable mental health profile.^14^ However, their reliance on self-reported symptoms does not allow for conclusions about the incidence of clinical levels of dysfunction and suffering. To our knowledge, there is no previous study with baseline data from birth and incidence of psychiatric disease as outcome. Therefore, the main aim of this study was to investigate the associations between parent-reported PA and time spent outdoors at 5, 8 and 11 years of age, participation in organised sports at 11 years of age and later incidence of psychiatric disease.

## Methods

### Study population

Our study analysed data from the ABIS Study (All Babies in Southeast Sweden), a prospective population-based birth cohort that includes data collected from 17 055 families with children born between 1 October 1997 and the 1 October 1999 in Southeast Sweden. The families included represent 78.6% of children born in the region, and thus provide diversity regarding sex, race, ethnicity, culture, socioeconomic conditions and marginalised groups, such as migrants and people with functional disabilities. Follow-ups during the first 4 years were made at well-baby clinics, which are free of cost for all families in Sweden. ABIS aims to investigate how environmental and genetic factors influence the development of immune-mediated diseases and the comorbidities that contribute to their burden, such as psychiatric disorders.^15^ Study participants have been followed up from birth onwards, and data have been collected up to 25 years of age. The parents were given oral, written and video information before giving their informed consent. The study was approved by the research ethics committees at Linköping University (Dnr 96–287, Dnr 99–321 and Dnr 03–092) and Lund University (LU 83–97) in Sweden. Connection to national registers was approved by the research ethics committee in Linköping (Dnr 2013/253-32).

### Equity, diversity, inclusion and patient involvement

The study included babies of all sexes, from all socioeconomic levels, participants with different ethnicities and from minority groups. Our methods of inclusion and data collection were the same for all participants. The author team consisted of three women and two men from different disciplines and with a wide range of experience in academia. Parents of study participants were involved in initial discussions about study design and appeared in videos used for information about the study. Parents were also involved in the construction of questionnaires.

### Diagnosis of psychiatric disorders

**Figure 1:**
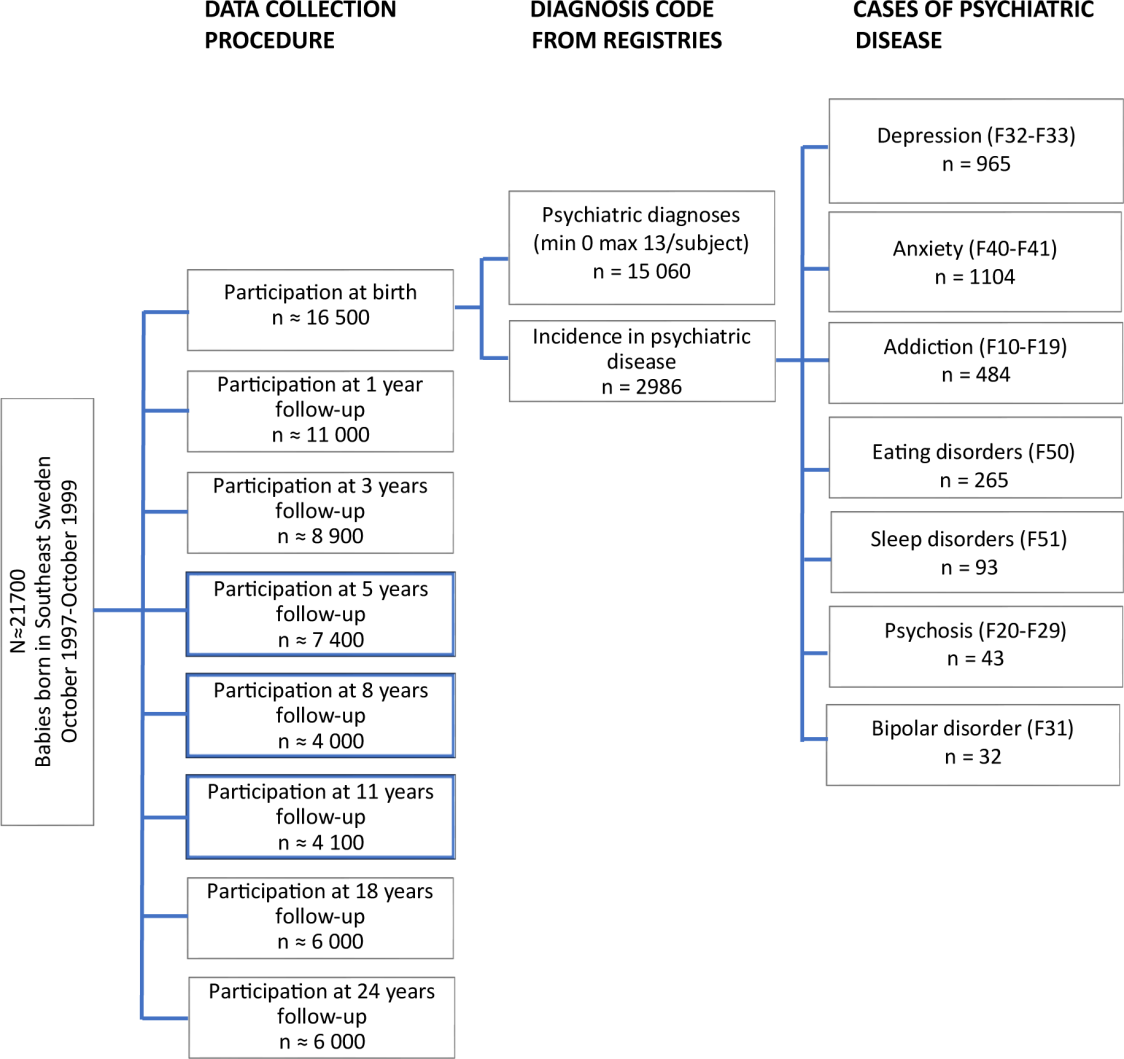
Study population flow chart. Definition of groups of cases based on the cumulative incidence rates for addictions, psychosis, depression, mania, anxiety, stress, eating and sleep disorders from birth until the age of 18 years.

Diagnoses (n=16 560) were obtained for the entire ABIS population from birth until 18 years of age, by cross-linking with the Swedish National Patient Register, containing all hospital inpatients (since 1973) and outpatients (since 2001) with the International Classification of Diseases (ICD-8 to ICD-10) diagnoses, made by physicians, in the majority of cases by specialists but also by general practitioners and paediatricians.^16^ The following ICD-10 diagnoses were obtained for the dataset: F10–F19 (addiction), F20–F29 (psychosis), F31 (bipolar disorder), F32–F33 (depression), F40–F41 (anxiety), F50 (eating disorders) and F51 (sleep disorders) (figure 1). Participants with neurodevelopmental disorders (autism F84, n=458 and attention deficit hyperactivity disorder F90, n=757) were excluded from the dataset since they are often diagnosed in early childhood, and it is debated whether they should be viewed as a normal variation in functioning.^17^ Learning difficulties (F70), language and other school difficulties (F80), conduct disorders (F91), tic disorders (F95) and personality disorders (F60) were excluded from our analyses, as an expert panel of child psychiatrists shared the view that the nature of these diagnoses differs from others, such as depression and anxiety disorders. The number of cases with psychoses (n=8) and bipolar disease (n=4), with valid PA data, was too few to allow reliable computations of hazard ratios. First-time incidences were used as events in the statistical analyses.

### Measures

Physical activity was reported through questionnaires filled out by parents when the child was 5, 8 and 11 years old. Parents were asked to respond to the questions: “How many hours per day on average is the child in motion (ie, playing, jumping or running around)?” and “How much time per day on average, does the child spend outdoors?” At 8 and 11 years, both questions were asked separately for school days and non-school days, which were weighted and calculated as average hours per day for PA and time spent outdoors. Participation in organised sports was reported in hours per week and measured only at age 11 years. Data on mothers’ use of psychotropics during pregnancy, and maternal education level at the birth of the child, classified according to the International Standard Classification of Education into three levels (low, medium and high, UNESCO 2012) and adverse life events at 5, 8 and 11 years, were obtained from parent-reported events in the questionnaires (online supplemental appendix 1).

### Statistical analyses

Characteristics of the study participants are presented as mean±SD or frequencies and percentages. Χ^2^ tests and t-tests were used to examine the cross-sectional associations. PA variables were used as continuous variables. Longitudinal associations between PA and psychiatric disorders were assessed with a two-way analysis of variance (ANOVA), in which interactions between PA and sex, maternal education, psychotropic use during pregnancy and adverse life events were analysed. Cox proportional hazard models were used to calculate hazard ratios (HRs) and their 95% CIs. Analyses were stratified by sex since we found a two-way interaction with PA (table 2), and adjustments were made for maternal education, use of psychotropics during pregnancy, as a proxy of mental health vulnerability, and adverse life events before 5, 8 and 11 years of age. All calculations of HR were prospective. Participants with psychiatric diagnoses, occurring before any of the three measurements, were excluded from analyses at the specific time points, since they would contribute to systematic bias and reversed causation could have been a problem. As psychiatric diagnoses are rare before the age of 11, this procedure resulted in a limited number of excluded subjects—for example, 4 for depression, 26 for anxiety and 2 for addiction at 11. At 5 and 8, even fewer cases were excluded. A total number of 192 statistical tests were conducted. Two-tailed tests with p values <0.05 were considered statistically significant. With a significance level of α=0.05, one would expect 10 significant associations by chance, compared with 43 reported in tables1^5^. Since we used two-sided testing, even though previous research could have supported a one-sided hypothesis, the risk of false positives on the beneficial tail of the distribution would have been 50% lower. Analyses were performed with the Statistical Package for the Social Sciences (SPSS 28, IBM, Armonk, New York, USA).

**Table 1:** Interaction effects for repeated measures of physical activity at 5, 8 and 11 years of age, in a two-way analysis of variance model with psychiatric diagnosis during childhood, sex, mothers’ education, psychotropic medication during pregnancy and adverse life event before 5 years of age

Two-way ANOVA model for repeated measures	Mean (SD) (hours/day)5 years	Mean (SD) (hours/day)8 years	Mean (SD) (hours/day)11 years	F	P value
PA (hours/day)					
Between subjects	4.2 (1.3)	3.6 (1.6)	2.5 (1.4)	223	< 0.01
Psychiatric diagnosis (yes/no)					
Between subjects	0.08 (0.27)	0.08 (0.27)	0.08 (0.27)	0.2	0.69
Sex (girl/boy)					
Between subjects	0.48 (0.5)	0.48 (0.5)	0.48 (0.5)	3.8	0.05
Mothers’ education (1–3)*					
Between subjects	2.17 (0.58)	2.17 (0.58)	2.17 (0.58)	3.8	0.02
Psychotropic medication					
Between subjects	0.01 (0.9)	0.01 (0.9)	0.01 (0.9)	0.1	0.82
Adverse life events (yes/no)					
Between subjects	0.20 (0.40)	0.26 (0.44)	0.31 (0.46)	3.8	0.05
**Interactions**					
Sex × PA					
Within subjects				3.4	0.04
Mothers’ education × PA					
Within subjects				1.2	0.33
Psychotropic med × PA					
Within subjects				2.8	0.06
Adverse life events × PA					
Within subjects				0.7	0.52

* Education stratified as (1) elementary school, (2) upper secondary school, (3) college and university.

ANOVA, analysis of variance; PA, physical activity.

**Table 5:** Cox proportional hazard models for time spent in organised sports at 11 years of age and incidence of psychiatric diagnoses during childhood

Organised sports (hours/week)	11 Years		
Diagnosis (ICD-10)	HR	95% CI	P value
Depression			
Girls			
Unadjusted	0.87	0.78 to 0.96	0.008
Adjusted	0.89	0.79 to 1.01	0.065
Boys			
Unadjusted	0.66	0.53 to 0.82	<0.001
Adjusted	0.65	0.51 to 0.84	<0.001
Anxiety			
Girls			
Unadjusted	0.82	0.74 to 0.92	<0.001
Adjusted	0.86	0.76 to 0.97	0.01
Boys			
Unadjusted	0.80	0.66 to 0.97	0.02
Adjusted	0.79	0.64 to 0.97	0.02
Addiction			
Girls			
Unadjusted	0.71	0.52 to0.98	0.04
Adjusted	0.59	0.39 to 0.89	0.01
Boys			
Unadjusted	0.72	0.59 to 0.89	0.002
Adjusted	0.70	0.56 to 0.88	0.002
Eating disorders			
Girls			
Unadjusted	1.00	0.88 to 1.15	0.98
Adjusted	0.94	0.79 to 1.11	0.47
Boys			
Unadjusted	1.51	0.70 to 3.27	0.30
Adjusted	1.81	0.79 to 4.17	0.16
Sleeping disorders			
Girls			
Unadjusted	0.73	0.46 to 1.16	0.18
Adjusted	0.73	0.45 to 1.17	0.19
Boys			
Unadjusted	0.93	0.48 to 1.78	0.82
Adjusted	1.09	0.57 to 2.07	0.80

Results are shown separately for girls and boys.

Adjusted for maternal education, psychotropic medication during pregnancy and adverse life events.

* Diagnoses include all psychotic, affective and stress-related diagnoses in ICD-10.

**Table 2:** Cox-proportional hazard models for physical activity at 5-, 8- and 11 years of age and incidence of any psychiatric disease* during childhood

Physical activity (hours/day)	5 Years			8 Years			11 Years		
Psychiatric disease1st diagnosis (n=1343)	HR	95% CI	P value	HR	95% CI	P value	HR	95% CI	P value
All participants									
Unadjusted	0.94	0.88 to 1.00	0.05	0.88	0.88 to 1.02	0.12	0.84	0.76 to 0.93	0.001
Adjusted	0.98	0.91 to 1.05	0.55	0.96	0.89 to 1.04	0.29	0.88	0.79 to 0.98	0.007
Girls									
Unadjusted	0.95	0.88 to 1.03	0.19	1.01	0.90 to 1.07	0.64	0.94	0.84 to 1.04	0.22
Adjusted	1.01	0.93 to 1.10	0.81	1.01	0.92 to 1.11	0.85	1.00	0.89 to 1.12	0.96
Boys									
Unadjusted	0.93	0.84 to 1.04	0.19	0.89	0.79 to 1.01	0.07	0.71	0.58 to 0.88	0.001
Adjusted	0.93	0.83 to 1.05	0.24	0.89	0.78 to 1.01	0.07	0.70	0.56 to 0.87	0.002

Results are shown for all participants and girls and boys separately.

Adjusted for maternal education, psychotropic medication during pregnancy and adverse life events.

* Diagnoses include all psychotic, affective and stress-related diagnoses in ICD-10.

**Table 3:** Cox-proportional hazard models for physical activity at 5, 8 and 11 years of age, and incidence of specific psychiatric diagnoses during childhood

Physical activity(hours/day)	5 Years			8 Years			11 Years		
Diagnosis (ICD-10)	HR	95% CI	P value	HR	95% CI	P value	HR	95% CI	P value
Depression									
Girls									
Unadjusted	0.95	0.83 to 1.07	0.37	0.93	0.80 to 1.07	0.28	0.75	0.62 to 0.90	0.002
Adjusted	1.00	0.87 to 1.15	0.99	0.96	0.82 to 1.12	0.62	0.82	0.67 to 1.00	0.05
Boys									
Unadjusted	0.81	0.68 to 0.96	0.01	0.82	0.65 to 1.03	0.08	0.69	0.48 to 0.99	0.05
Adjusted	0.81	0.67 to 0.98	0.03	0.77	0.59 to 0.99	0.05	0.71	0.47 to 1.06	0.09
Anxiety									
Girls									
Unadjusted	0.96	0.86 to 1.07	0.47	1.08	0.94 to 1.24	0.28	0.92	0.78 to 1.08	0.30
Adjusted	1.02	0.90 to 1.16	0.76	1.09	0.89 to 1.32	0.41	0.96	0.81 to 1.13	0.61
Boys									
Unadjusted	0.81	0.66 to 0.99	0.04	0.91	0.71 to 1.15	0.43	0.67	0.47 to 0.95	0.02
Adjusted	0.79	0.63 to 0.98	0.03	0.86	0.61 to 1.22	0.41	0.61	0.42 to 0.90	0.01
Addiction									
Girls									
Unadjusted	1.14	0.84 to 1.55	0.40	0.76	0.52 to 1.12	0.16	0.93	0.61 to 1.42	0.75
Adjusted	1.16	0.83 to 1.61	0.39	0.80	0.54 to 1.18	0.26	1.04	0.68 to 1.58	0.87
Boys (n=105)									
Unadjusted	1.12	0.89 to 1.41	0.33	0.76	0.60 to 0.97	0.02	0.65	0.46 to 0.91	0.01
Adjusted	1.16	0.90 to 1.49	0.24	0.76	0.59 to 0.98	0.03	0.65	0.45 to 0.95	0.02
Eating disorders									
Girls (n=166)									
Unadjusted	0.99	0.84 to 1.17	0.93	0.99	0.83 to 1.18	0.93	0.93	0.74 to 1.16	0.51
Adjusted	1.21	0.98 to 1.48	0.76	1.06	0.87 to 1.29	0.57	1.06	0.83 to 1.36	0.63
Boys									
Unadjusted	1.36	0.67 to 2.76	0.40	1.38	0.82 to 2.34	0.23	1.61	0.70 to 3.69	0.26
Adjusted	1.42	0.63 to 3.23	0.40	1.31	0.77 to 2.22	0.32	1.36	0.63 to 2.94	0.43
Sleep disorders									
Girls									
Unadjusted	1.56	0.93 to 2.62	0.09	0.59	0.27 to 1.29	0.19	1.01	0.56 to 1.83	0.97
Adjusted	1.53	0.91 to 2.57	0.11	0.58	0.27 to 1.27	0.18	0.99	0.55 to 1.78	0.96
Boys									
Unadjusted	1.36	0.76 to 2.42	0.30	0.81	0.41 to 1.59	0.54	0.66	0.19 to 2.27	0.51
Adjusted	1.32	0.73 to 2.37	0.36	0.77	0.39 to 1.50	0.44	0.66	0.22 to 1.96	0.46

Results are shown separately for girls and boys.

Adjusted for sex (except when the analyses were stratified by sex), maternal education, psychotropic medication during pregnancy and adverse life events.

* Diagnoses include all psychotic, affective and stress-related diagnoses in ICD-10.

**Table 4:** Cox proportional hazard models for time in organised sports at 11 years of age, and incidence of any psychiatric disease* during childhood

Organised sports (hours/week)	11 Years		
Psychiatric disease1st diagnosis	HR	95% CI	P value
All participants			
Unadjusted	0.84	0.79 to 0.89	<0.001
Adjusted	0.85	0.79 to 0.91	<0.001
Girls			
Unadjusted	0.88	0.82 to 0.94	<0.001
Adjusted	0.88	0.82 to 0.96	0.003
Boys			
Unadjusted	0.78	0.69 to 0.87	<0.001
Adjusted	0.77	0.68 to 0.88	<0.001

Results are shown for all participants and girls and boys separately.

Models adjusted for sex (except when the analyses were stratified by sex), maternal education, psychotropic medication during pregnancy and adverse life events.

* Diagnoses include all psychotic, affective and stress-related diagnoses in ICD-10.

## Results

### Descriptive statistics

Of the 16 365 children included in the study at birth, 7880 (48%) were girls and 8485 (52%) were boys. Twenty-seven per cent of the participants’ mothers had a university degree and less than 0.1% reported that psychotropic medicines were taken during pregnancy. Twenty per cent reported an adverse event before 5 years of age and almost 30% at 10 years. A total of 1353 participants (15%) were diagnosed with at least one psychiatric diagnosis during childhood (table 6), and 4% had three or more diagnoses. PA continually declined between 5, 8 and 11 years of age, but there were no significant differences between girls and boys (table 6). According to the parents, girls and boys spent the same amount of time outdoors. Boys were reported to spend more time participating in organised sports (3.3 hours/week vs 3.0 hours/week, p<0.01) at 11 years of age.

**Table 6:** Demographics and descriptions of study variables

	All (n=16 365)	Girls (n=7880)	Boys (n=8485)	P value*
Maternal education				
High school (%)	73.3	72.8	73.7	0.709
University degree (%)	26.7	27.2	26.3	0.242
Psychotropic medicine during pregnancy				
Yes/no	135/16 365	65/7880	70/8485	0.999
Adverse life events (% of total)				
Before 5 years	2491 (15)	1148 (15)	1343 (16)	0.019
Before 8 years	3326 (20)	1518 (19)	1808 (21)	0.001
Before 11 years	4108 (25)	1906 (24)	2202 (26)	0.007
Diagnoses 0–18 year, n (%) of total conditions†				
Any diagnosis	1353 (100)	878 (65)	475 (35)	<0.001
Mean age (SD), years	15.4 (2.4)	15.6 (2.1)	15.1 (2.9)	<0.001
Depression‡	497 (37)	333 (38)	164 (35)	<0.001
Mean age (SD)	16.0 (1.4)	16.0 (1.4)	15.9 (1.5)	<0.001
Anxiety	570 (42)	410 (47)	160 (34)	<0.001
Mean age (SD)	15.8 (1.8)	15.9 (1.6)	15.3 (2.2)	<0.001
Addiction	183 (14)	78 (9)	105 (22)	<0.001
Mean age (SD)	16.4 (1.3)	16.3 (1.2)	16.4 (1.3)	0.159
Eating disorders	183 (14)	166 (12)	17 (1)	<0.001
Mean age (SD)	15.0 (2.0)	15.2 (1.8)	13.5 (2.8)	<0.001
Sleep disorders	58 (4)	30 (2)	28 (2)	<0.001
Mean age (SD)	12.5 (5.7)	14.0 (5.1)	10.9 (6.1)	0.093
Number of diagnoses§				
% of population				
0 diagnosis	85	81	89	
1 diagnosis	8	9	7	
2 diagnoses	3	4	2	
3 or more diagnoses	4	6	2	
Physical activity				
hours/day, mean (SD)				
At 5 years of age	4.2 (1.3)	4.2 (1.3)	4.2 (1.3)	0.145
(n=)¶	7305	3487	3818	
At 8 years of age	3.6 (1.6)	3.5 (1.7)	3.6 (1.6)	0.264
(n=)	3942	1864	2078	
At 11 years of age	2.5 (1.4)	2.4 (1.3)	2.7 (1.3)	<0.001
(n=)	3890	1903	1987	
Time outdoors				
hours/day, mean (SD)				
At 5 years of age	3.5 (2.0)	3.4 (2.0)	3.6 (2.0)	<0.001
(n=)¶	7320	3506	3814	
At 8 years of age	1.9 (1.0)	1.9 (1.0)	2.0 (1.0)	<0.001
(n=)	3977	1883	2094	
At 11 years of age	1.0 (0.9)	1.0 (0.9)	1.0 (0.9)	<0.001
(n=)	4024	1985	2039	
Organised sports				
hours/week, mean (SD)				
At 11 years of age	3.2 (2.1)	3.0 (2.1)	3.3 (2.1)	<0.001
(n=)	4070	2012	2058	

* Differences between girls and boys were calculated with t-test (means) and Χ2 (frequencies).

† Total conditions is the same as any 1st time incidence of a condition (please see Methods section for diagnoses included).

‡ Difference between groups regarding diagnoses not calculated since they were zero at baseline.

§ Psychiatric (ICD-10).

¶ n=number of participants with valid physical activity data.

### Interactions

table 1 shows results from two-way ANOVA analyses of interactions. There was a significant two-way interaction for sex (F 3.4, p=0.04, online supplemental figure S.1), and this finding provided the rationale for separating the results for girls and boys in the Cox proportional hazard models used to calculate hazard ratios for incidence of any, and specific, psychiatric diseases.

### PA and incidence of psychiatric conditions

With full adjustment for potential confounders, PA was associated with the first-time incidence of any psychiatric disease for boys at 11 years of age (HR=0.70, 95% CI 0.56 to 0.87) but not for girls (table 2). A trend in this direction could also be seen at 8 years for boys (HR=0.89, 95% CI 0.78 to 1.01). When the incidence of specific psychiatric diagnoses was analysed in relationship to daily PA (table 3), our results showed a trend towards decreased risk for depression for girls (HR=0.82, 95% CI 0.67 to 1.00) and a trend for boys (HR=0.71, 95% CI 0.47 to 1.06), at 11 years of age. For boys, but not for girls, a decreased risk of depression was shown at 5 years (HR=0.81, 95% CI 0.67 to 0.98) and 8 years (HR=0.77, 95% CI 0.59 to 0.99) of age. There was a marked decreased risk for anxiety among boys, but not for girls, at both 5 (HR=0.79, 95% CI 0.63 to 0.98) and 11 years (HR=0.61, 95% CI 0.42 to 0.90). The risk of addiction was lower for boys at 8 (HR=0.76, 95% CI 0.59 to 0.98) and 11 years (HR=0.65, 95% CI 0.45 to 0.95) of age, but not for girls (table 3).

### Time outdoors and incidence of psychiatric conditions

There were no associations between parent-reported time spent outdoors and the incidence of first-time psychiatric disease, or with incidence in specific psychiatric diagnoses (online supplemental tables S.1 and S.2).

### Participation in organised sports and incidence of psychiatric conditions

The weekly amount of participation in organised sports at 11 years of age was associated with a decreased risk for first-time incidence of any psychiatric conditions for both girls (HR=0.88, 95% CI 0.82 to 0.96) and boys (HR=0.77, 95% CI 0.68 to 0.88) (table 4). When the incidence of specific psychiatric diagnoses was analysed in relationship to organised sports (table 5), there was a decreased risk for incidence of depression for boys, but only a trend for girls (boys: HR=0.65, 95% CI 0.51 to 0.84, girls: HR=0.89, 95% CI 0.79 to 1.01). There was a lower risk for anxiety among both girls and boys (girls: HR=0.86, 95% CI 0.76 to 0.97, boys: HR=0.79, 95% CI 0.64 to 0.97) and a decreased risk for addiction (girls: HR=0.59, 95% CI 0.39 to 0.89, boys: HR=0.70, 95% CI 0.56 to 0.88). There were no changes in the risk for eating disorders or sleep disorders.

## Discussion

This study aimed to analyse if parent-reported amounts of PA, time outdoors and participation in organised sports in early childhood were associated with the incidence of psychiatric disorders until 18 years of age. The incidence rate in our study population of 15% is in accordance with meta-analytic evidence of global incidence rates of 10–20%.^18^ Our results showed that the daily amount of PA at 11 years of age was associated with a 12% decreased risk for every hour of PA (HR=0.88, p=0.007), of being diagnosed with any psychiatric disease before 18 years of age. These associations were mainly driven by the effects on boys. These results should be viewed considering the steep decline in daily PA between the three time points, which highlights a problematic trend towards increased inactivity during this period (table 6).

However, when our analyses included specific psychiatric diagnoses as outcomes, we found the effects of PA already at 5 years of age. Thus, the risk for subsequent diagnosis of anxiety disorder was sharply decreased (HR=0.61, p=0.01) for boys at both 5 and 11 years of age, but not at 8, but there was no preventive effect for girls. The same overall pattern could be observed for the incidence of addiction, in which boys but not girls had a protective effect from PA. Except for the indication of a significant preventive effect for the incidence of depression at both 5 and 8 years of age for boys, there were no significant associations for any of the outcomes of the other specific diagnoses (table 3). At 11 years of age, PA was associated with a clear trend for decreased risk for depression among both girls and boys.

Our results are in line with findings from Wu *et al*,^19^ who in a study of 4861 Canadian 10–11-year-old children, showed that the incidence of internalising and externalising psychiatric conditions was higher among less physically active children. To our knowledge, that study is the only previous prospective study of PA and psychiatric disease among children and youth with diagnoses as outcomes.

Our results confirm these earlier findings and extend them by also reporting the incidence of specific diagnoses. Notably, our results revealed clear sex differences—for example, a significant protective effect of PA on anxiety and addiction for boys but not for girls at 11 years of age. Differences such as these have been reported earlier, including by Isaksson *et al*,^20^ who showed a protective effect against depression among boys but not girls in a cohort of 14-year-old children. Physical activity may influence boys and girls in different ways, including changes mediated by different levels of sex hormones. However, these effects might be direct—for example, by influencing brain health and development, or indirect—for example, by reducing subclinical hyperactivity, which is more common among boys. Furthermore, there may exist differences in physiological consequences (eg, variation in intensity of PA) and psychological experiences, that are connected to gender and cultural norms. Future studies should investigate these mediating and modifying factors.

For the weekly amount of engagement in organised sports, which in Sweden means activities in sports clubs, we found support for strong protective effects for both girls and boys on the risk for depression, anxiety and addiction (tables4  5.). This is in line with earlier research, in particular of team sports,^21^ in which less pressure is on the individual and the social component represents a multifaceted environmental enrichment.^22^ While it is notable that 11-year-old boys had a decreased risk of subsequent depression by almost 50% for every extra hour per week of participation in organised sports, it should be taken into consideration that already present subclinical social and psychological difficulties might have influenced their level of sports participation.^23^ In our analyses, we adjusted for factors related to vulnerability, but not for baseline well-being.

Our results support the hypothesis that the period just before, and during, the early stages of puberty might represent a sensitive period in which PA is key to the development of resilience and hardiness. Indeed, we know from epidemiological studies and neurobiological data, that this period is situated at the peak of anxiety disorder incidence, at the time when affective disorders become more common and may correspond to a period in which myelination of central nervous system neurons peak and the pruning of synapses begin.^24^ Our results suggest that the age 10–12 represents a window of opportunity to cultivate resilience. Our findings do not imply that daily movement is less important during earlier periods of childhood, where it might catalyse the healthy development of several physical, cognitive and social skills.^25^

The results from our study do not allow conclusions to be reached about mechanisms involved in the relationships between PA and the development of psychopathology. Evidence for the hypothesis that dysregulated inflammation plays a key role in the development of psychiatric symptoms^26^ has grown, but knowledge about molecular pathways has not had much of an impact on clinical work or public health policies. Perhaps PA might be the most effective way of translating knowledge of biological mechanisms into practical interventions.

### Limitations

Our results should be interpreted with caution, and conclusions should be drawn with several limitations in mind. First, all exposure variables relied on parents reporting daily or weekly levels of PA, time spent outdoors and participation in organised sports. This method is less precise than objective measures and could be vulnerable to recall bias. Furthermore, items were not consistent at the various time points, but we believe they were adapted to changes in the way in which children in different ages are physically active and that our calculations of hours per day or week make the measurements comparable. Overall, we have no reason to believe that the registration of PA in this study was biased in a way that influences the associations we have found. A more robust method of PA assessment is the use of accelerometers.^27^ Although objective and more precise, accelerometry has limitations, such as influencing the subject (eg, inspiration to move) and only providing a snapshot (eg, 7 days), which does not account for the temporal variability in children’s levels of activity.^27^

Second, data on the incidence of psychiatric diseases was obtained from a national registry.

Third, the ambition to investigate incidence in specific diagnosis resulted in a rather large number of statistical tests. However, the number of statistically significant associations was more than fourfold those expected by chance alone.

Fourth, it is important to recognise that the data were collected in a country with plenty of resources. Thus generalisability to settings with fewer resources, such as low- and middle-income countries, may be somewhat difficult.

### Strengths

The study also has several strengths. The registry has been shown to have a high coverage of births and diagnoses,^16^ but many children with mild mental health issues never receive a diagnosis. The use of the incidence of psychiatric disease is a strength, since temporary psychosocial suffering will not be diagnosed as a clinical syndrome, while it might have resulted in a high score on a self-report questionnaire. Our prospective data, and our analyses without any diagnoses occurring before the PA exposure, largely prevent reverse causation. However, our analyses do not consider subclinical levels of dysfunction, which means that results have a crudeness, and future studies should also include dimensionality.^28^ This study has several other strengths, and the large birth cohort with whole-childhood follow-up is notable. Another strength is the multiple measurements of PA, which allowed the study of interactions, and enabled us to observe trends (eg, the clustering of effects at the 11-year-time point) and generate hypotheses about a potential critical period before and during the beginning of adolescence.

### Implications

The results of this study could inform public health professionals concerned with trends of increased prevalence of mental health disorders and the alarming fact that many children around the world do not meet the recommendations for PA.^29^ A growing body of evidence for the broad beneficial effects of daily movement, and associated social contexts, could inform policymakers’ decisions about the inclusion of more PA in the school curriculum. Furthermore, our data could specify during which years PA should have priority. From a broader perspective, political initiatives, motivated by studies such as ours, should remove barriers to daily exercise, such as safe manual transportation and equal access to environments that invite an active way of living.^30^ The results could also be of interest to mental health professionals, supporting their inclusion of PA and organised sports in both preventive initiatives and in the treatment of psychiatric conditions. The field of mental health needs more effective interventions for the treatment of psychiatric disease, and future studies should evaluate novel ways to include PA in the treatment of children and youth. Future research should investigate PA as both a stand-alone treatment and as a complement to other evidence-based interventions.

In conclusion, this study showed that PA, and in particular, participation in organised sports, may provide sex-specific protective effects against future incidence of several childhood psychiatric diseases. The results remained after adjustment for several confounding factors and support the hypothesis that the pre-adolescent period might be a critical period for the development of psychological resilience.

## Supplementary material

10.1136/bjsports-2024-108148 online supplemental file 1

## Data Availability

Data are available upon reasonable request.
