# Supplementary material for: Impact of physical activity on the incidence of psychiatric conditions during childhood: a longitudinal Swedish birth cohort study
Source: Br J Sports Med. 2025 May 13;59(14):e108148. doi: 10.1136/bjsports-2024-108148 (PMC12229077; doi:10.1136/bjsports-2024-108148)
Supplement: online supplemental file 1 [file bjsports-59-14-s001.docx]

**Supplemental figures, tables and appendices**

**Figure S.1** **Mean physical activity among boys and girls at 5-, 8-, and 11 years**

**Figure 2: Mean physical activity among boys and girls at 5-, 8-, and 11 years of age**

**
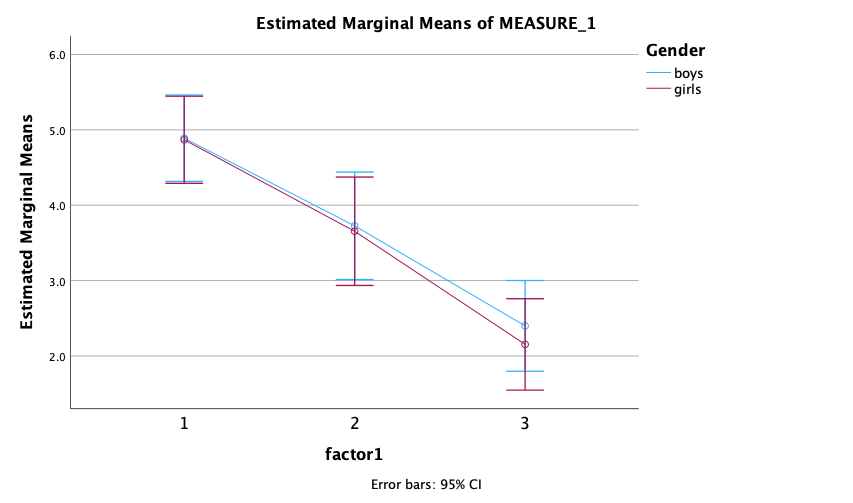
**

**Hours/day 5-years 8-years 11-years**

Error bars represent 95 % confidence interval.

**Table S.1 Cox-proportional hazard models for time outdoors at 5-, 8- and 11 years of age and incidence of any psychiatric disease* during childhood. Results are shown for all participants and girls and boys separately.**

| **Time outdoors**  (hours/day) |  | **5 Years** |  |  | **8 Years** |  |  | **11 Years** |  |
| --- | --- | --- | --- | --- | --- | --- | --- | --- | --- |
| **Psychiatric disease**  1:st diagnosis  (n=1343) | HR | CI (95 %) | *p-*Value | HR | CI (95 %) | *p*-Value | HR | CI (95 %) | *p*-Value |
|  |  |  |  |  |  |  |  |  |  |
| **All participants** |  |  |  |  |  |  |  |  |  |
| Unadjusted | 0.99 | 0.99-0.95 | 0.63 | 1.03 | 0.92-1.15 | 0.64 | 0.94 | 0.92-1.10 | 0.40 |
| Adjusted | 1.01 | 0.96-1.05 | 0.818 | 1.08 | 0.96-1.21 | 0.22 | 1.01 | 0.87-1.17 | 0.93 |
| **Girls** |  |  |  |  |  |  |  |  |  |
| Unadjusted | 1.03 | 0.98-1.08 | 0.26 | 1.10 | 0.96-1.27 | 0.18 | 1.01 | 0.86-1.18 | 0.89 |
| Adjusted | 1.07 | 0.97-1.19 | 0.19 | 1.15 | 0.99-1.34 | 0.06 | 1.08 | 0.92-1.27 | 0.36 |
| **Boys** |  |  |  |  |  |  |  |  |  |
| Unadjusted | 0.95 | 0.88-1.02 | 0.16 | 0.97 | 0.80-1.18 | 0.76 | 0.89 | 0.67-1.18 | 0.42 |
| Adjusted | 0.92 | 0.80-1.06 | 0.26 | 0.96 | 0.78-1.18 | 0.69 | 0.81 | 0.58-1.13 | 0.22 |

Models adjusted for sex (all participants) maternal education, psychotropic medication during pregnancy and adverse life events.

* Diagnoses include all psychotic, affective, and stress-related diagnoses in ICD-10.

**Table S.2 Cox-proportional hazard models for time outdoors at 5-, 8-, and 11 years of age and incidence of psychiatric diagnoses during childhood. Results are shown separately for girls and boys.**

| **Time outdoors**  (hours/day) |  | | **5 Years** | |  |  | | **8 Years** | |  |  | | **11 Years** |  |
| --- | --- | --- | --- | --- | --- | --- | --- | --- | --- | --- | --- | --- | --- | --- |
| **Diagnosis**  (ICD-10) | HR | CI (95 %) | | *p*-Value | | HR | CI (95 %) | | *p*-Value | | HR | CI (95 %) | | *p*-Value |
| **Depression** |  | |  | |  |  | |  | |  |  | |  |  |
| **Girls** |  | |  | |  |  | |  | |  |  | |  |  |
| Unadjusted | 1.03 | | 0.95-1.12 | | 0.45 | 1.10 | | 0.86-1.35 | | 0.53 | 0.91 | | 0.69-1.19 | 0.49 |
| Adjusted | 1.06 | | 0.97-1.16 | | 0.20 | 1.15 | | 0.90-1.46 | | 0.27 | 0.99 | | 0.75-1.30 | 0.99 |
| **Boys** |  | |  | |  |  | |  | |  |  | |  |  |
| Unadjusted | 0.81 | | 0.71-0.93 | | 0.003 | 0.73 | | 0.50-1.10 | | 0.12 | 0.72 | | 0.58-1.46 | 0.72 |
| Adjusted | 0.83 | | 0.72-0.96 | | 0.01 | 0.68 | | 0.44-1.06 | | 0.09 | 0.71 | | 0.38-1.34 | 0.29 |
| **Anxiety** |  | |  | |  |  | |  | |  |  | |  |  |
| **Girls** |  | |  | |  |  | |  | |  |  | |  |  |
| Unadjusted | 1.05 | | 0.98-1.13 | | 0.17 | 1.09 | | 0.88-1.38 | | 0.41 | 1.01 | | 0.80-1.27 | 0.97 |
| Adjusted | 1.05 | | 0.96-1.13 | | 0.30 | 1.11 | | 0.88-1.41 | | 0.37 | 1.02 | | 0.80-1.31 | 0.87 |
| **Boys** |  | |  | |  |  | |  | |  |  | |  |  |
| Unadjusted | 0.96 | | 0.85-1.10 | | 0.59 | 1.00 | | 0.70-1.43 | | 0.99 | 0.53 | | 0.25-1.11 | 0.09 |
| Adjusted | 0.93 | | 0.81-1.07 | | 0.30 | 0.94 | | 0.65-1.36 | | 0.74 | 0.45 | | 0.20-1.02 | 0.06 |
| **Addiction** |  | |  | |  |  | |  | |  |  | |  |  |
| **Girls** |  | |  | |  |  | |  | |  |  | |  |  |
| Unadjusted | 0.98 | | 0.80-1.19 | | 0.82 | 0.86 | | 0.46-1.64 | | 0.65 | 0.97 | | 0.55-1.88 | 0.97 |
| Adjusted | 0.95 | | 0.76-1.17 | | 0.62 | 0.87 | | 0.45-1.70 | | 0.68 | 1.09 | | 0.60-1.98 | 0.78 |
| **Boys** |  | |  | |  |  | |  | |  |  | |  |  |
| Unadjusted | 1.01 | | 0.88-1.17 | | 0.88 | 0.83 | | 0.56-1.21 | | 0.33 | 0.97 | | 0.63-1.51 | 0.97 |
| Adjusted | 1.01 | | 0.87-1.17 | | 0.92 | 0.84 | | 0.57-1.25 | | 0.40 | 1.03 | | 0.67-1.58 | 0.91 |
| **Eating disorders** |  | |  | |  |  | |  | |  |  | |  |  |
| **Girls** |  | |  | |  |  | |  | |  |  | |  |  |
| Unadjusted | 1.03 | | 0.93-1.15 | | 0.59 | 1.17 | | 0.89-1.53 | | 0.26 | 0.82 | | 0.55-1.24 | 0.35 |
| Adjusted | 1.09 | | 0.96-1.22 | | 0.17 | 1.27 | | 0.95-1.69 | | 0.11 | 0.93 | | 0.61-1.44 | 0.76 |
| **Boys** |  | |  | |  |  | |  | |  |  | |  |  |
| Unadjusted | 1.33 | | 0.91-1.95 | | 0.14 | 1.46 | | 0.75-2.83 | | 0.27 | 1.59 | | 0.79-3.18 | 0.19 |
| Adjusted | 1.48 | | 0.97-2.26 | | 0.07 | 1.31 | | 0.66-2.59 | | 0.44 | 1.59 | | 0.70-3.60 | 0.26 |
| **Sleep disorders** |  | |  | |  |  | |  | |  |  | |  |  |
| **Girls** |  | |  | |  |  | |  | |  |  | |  |  |
| Unadjusted | 0.87 | | 0.64-1.19 | | 0.39 | 0.86 | | 0.28-2.62 | | 0.80 | 1.01 | | 0.45-2.47 | 0.89 |
| Adjusted | 0.88 | | 0.64-1.21 | | 0.43 | 0.86 | | 0.28-2.65 | | 0.79 | 1.07 | | 0.45-2.55 | 0.88 |
| **Boys** |  | |  | |  |  | |  | |  |  | |  |  |
| Unadjusted | 0.86 | | 0.59-1.26 | | 0.43 | 0.49 | | 0.14-1.75 | | 0.27 | 0.92 | | 0.18-4.68 | 0.92 |
| Adjusted | 0.84 | | 0.57-1.22 | | 0.36 | 0.45 | | 0.13-1.52 | | 0.20 | 0.73 | | 0.11-4.68 | 0.74 |

Models adjusted for maternal education, psychotropic medication during pregnancy and adverse life events.

* Diagnoses include all psychotic-, affective-, and stress-related diagnoses in ICD-10.

**Appendix 1: Variable description**

| **Measurements** | **Physical activity** | **Adverse life events** | **Confounders** |
| --- | --- | --- | --- |
| **0-5 years**  **(3 occasions)** | (3) How much time does the child spend outdoors, on average, per day? (Less than 30m, 30m-1h, 1-2h, 2-4h, 4-6h, 6-8h, more than 8h).  (5) How many hours a day is your child in motion (playing, jumping, running around) on average? (never, 0-30m, 30m-1h, 1-2h, 2-3h, 3-4h, 4-5h, 5-6h, 7 or more).  (5) On average, how much time does the child spend outdoors per day? (less than 30m, 30m-1h, 1-2h, 2-3h, 3-4h, 5-6h, 7-8h). | Longitudinal/composite measurement of serious life events from 1 to 11 years of age. INCLUDED children or family stress (when was not possible to separate):  b_44a: Difficult life event (yes, no) / b_44txt What difficult life event?  c_30a: Has the child or family been involved in a serious or dramatic incident (death, divorce, new care giver etc.)? (yes, no) / c_30atx: If yes, what kind of incident?  e_108: Has the child been exposed to, what you consider to be, a difficult life event since birth?  A relative died / Parents who divorced / A new adult in the family / One or more new children in the family (a new biological sibling does not count) / Many conflicts between adults in the home / Contact with a supporting family / Support measure from social authorities (not social welfare) / Foster care placement / Other difficult life event (txt).  f_82a: Has the child been exposed to, what you consider to be, a difficult life event in the past 2 years?  A parent or a sibling died / A grandparent died / The child has been / is seriously ill / Serious illness in the family (not the child) / Many conflicts between adults in the home / Parents who divorced / Shared custody (alternating housing) / Sole custody (regular contact with the other parent) / Sole custody (sporadic or no contact with the other parent) / New adults in the family / New child-children in the family / Contact with a supporting family / Support measure from social authorities (not social welfare) / Foster care placement / Other difficult life event / What other difficult life event? (txt)  gv_64: Has the child been involved in a difficult life event in the last 2 years?  Parent or sibling who died / Grandparent who died / The child has been/is seriously ill / Serious illness in the family (except the child) / Many conflicts between adults in the home / Divorced/separated parents / Shared custody (lives alternately with mother and father) / Sole custody (regular contact with the other parent) / Sole custody (no or only sporadic contact with the other parent) / New adults in the family / New child(ren) in the family(s) (also "bonus siblings") / Contact with support family / Support measures from social authorities (social benefits are not included) / Foster home placement. Other difficult life event (txt). | Sex  Did the mother use psychotropics during your pregnancy (yes, no, do not know)  Parents born in Sweden (yes,no)  Parental education level (low, medium, high) |
| **8 years** | How many hours a day is your child in motion (playing, jumping, running around) on average? School day (0-15m, 30m, 1h, 2h, 3h, 4h, 5h, 6h, 7 or more)  How many hours a day is your child in motion (playing, jumping, running around) on average? Not a school day (0-15m, 30m, 1h, 2h, 3h, 4h, 5h, 6h, 7 or more)  How much time a day does the child spend outside on average? School day (0-15m, 30m, 1h, 2h, 3h, 4h, 5h, 6h, 7 or more)  How much time a day does the child spend outside on average? Not a school day (0-15m, 30m, 1h, 2h, 3h, 4h, 5h, 6h, 7 or more)  What does the child usually do during breaks? (playing indoor, playing outdoors, sitting still ex reading)  Are the students allowed to stay inside during breaks? (yes,no)  How many lesson hours does the child have scheduled sports / gymnastics per week? (1, 2, 3, 4, 5, 6, 7 or more)  How many hours per week (not during school time) does the child have organized physical activity (soccer, dance, floorball, riding etc.)? (1, 2, 3, 4, 5, 6, 7 or more) |  |  |
| **11 years** | (C) What do you usually do during breaks? Be still (for example, sit and talk or read), Being in motion (for example playing, walking, playing football).  (C) How many days a week do you have gymnastics / sports at school? (0, 1, 2, 3, 4, 5)  (P) How many hours per day on average is the child in motion (playing, jogging, running around)? School day (not at all, about half an hour, about 1 hour, about 2 hours, about 3 hours, about 4 hours, about 5 hours, about 6 hours, about 7 hours or more)  (P) How many hours per day on average is the child in motion (playing, jogging, running around)? Not school day (not at all, about half an hour, about 1 hour, about 2 hours, about 3 hours, about 4 hours, about 5 hours, about 6 hours, about 7 hours or more)  (P) How much does the child spend outdoors on average per day? School day (not at all, about half an hour, about 1 hour, about 2 hours, about 3 hours, about 4 hours, about 5 hours, about 6 hours, about 7 hours or more)  (P) How much does the child spend outdoors on average per day? Not school day (not at all, about half an hour, about 1 hour, about 2 hours, about 3 hours, about 4 hours, about 5 hours, about 6 hours, about 7 hours or more)  (P) How many hours per week during leisure time (not school time), does the child have organized physical activity (eg football, dancing, floorball, horse riding, scouts, etc)? (not at all, about half an hour, about 1 hour, about 2 hours, about 3 hours, about 4 hours, about 5 hours, about 6 hours, about 7 hours or more) |  |  |

**(C) = Children reported, (P) = Parent reported.**
